# Escore de Risco Clínico Simples para Prever a Mortalidade Pós Alta Hospitalar em Pacientes Chineses Hospitalizados por Insuficiência Cardíaca

**DOI:** 10.36660/abc.2022050

**Published:** 2023-02-13

**Authors:** Guillermo Alberto Arana Morales, Hugo Alpaca-Salvador, Ricardo Salazar-Ramírez

**Affiliations:** 1 Universidade Nacional de Santa Chimbote Peru Universidade Nacional de Santa, Chimbote - Peru

Lemos com grande interesse o artigo de Wang et al.,^1^ sobre o escore de risco clínico para prever mortalidade em pacientes chineses hospitalizados por insuficiência cardíaca (IC). Os autores desenvolveram uma escala com cinco variáveis para prever mortalidade por IC um ano após a alta hospitalar: idade, sexo feminino, pontuação na escala da New York Heart Association (NYHA) >3, diâmetro do átrio esquerdo e índice de massa corporal; com boa capacidade preditiva.^1^ Uma limitação do estudo é o método de divisão aleatória em dois grupos, um para o desenvolvimento do modelo preditivo e outro para avaliar seu desempenho, que, quando extraídos da mesma coorte, têm características semelhantes e, portanto, o desempenho preditivo pode ser superestimado.^2^

Um aspeto muito interessante do estudo é que ele se concentra na avaliação de pacientes com IC na alta hospitalar, utiliza variáveis preditivas simples facilmente avaliáveis e prevê a mortalidade em um ano. Há uma variedade de estudos de escores prognósticos para IC, em pacientes ambulatoriais, hospitalizados ou de emergência e preveem mortalidade durante a internação, nos primeiros 30 dias ou um ano; muitos deles com vieses que limitam sua validez.

Uma revisão sistemática avaliou escores prognósticos em pacientes com IC aguda na sala de emergência. Oito estudos foram identificados. O resultado preditivo mais frequentemente estimado foi mortalidade em 30 dias. Os escores avaliaram os seguintes preditores: idade, classe NYHA, pressão sistólica, pressão diastólica, saturação de oxigênio, pCO_2_ (pressão parcial de dióxido de carbono), creatinina, e peptídeo natriurético atrial. Apenas duas escalas avaliadas foram muito precisas e devidamente validadas.^3^

Iwakami et al. avaliaram o desempenho de escores prognósticos existentes em uma coorte de pacientes japoneses com IC aguda para prever mortalidade em 30 dias. Avaliaram as características do estudo de referência e aplicou as ferramentas para determinar o viés. Dos 6340 artigos identificados, eles estudaram 224 escores. Apenas 30 (13%) relataram c-estatística na coorte de derivação. Quando os modelos identificados foram aplicados à coorte japonesa, foi encontrada uma estatística-c geral de 0,64. O estudo encontrou uma boa correlação entre o baixo risco de viés na seleção da amostra e a estatística-c. Concluíram que uma amostra ótima no estudo de derivação é fundamental para determinar o desempenho dos escores prognósticos de IC.^4^

O modelo preditivo proposto por Wang et al.,^1^ ou outros modelos, podem ser aplicados a pacientes da população latino-americana? Sprockel et al.,^5^ avaliaram três modelos preditivos existentes para estimar a morte intra-hospitalar em pacientes com IC aguda em um hospital na Colômbia; foram avaliados os modelos ADHERE, OPTIMIZE-HF e GWTG-HF. O estudo conclui que os escores de risco avaliados apresentaram baixa capacidade de discriminar o risco de mortalidade hospitalar.^5^

Estudos de validade externa na América Latina são definitivamente necessários para avaliar o desempenho, estabilidade e reprodutibilidade de modelos preditivos aplicados em outras áreas geográficas ou, melhor ainda, realizar estudos para desenvolver e validar seus próprios modelos.

Palavras-chave: Insuficiência Cardíaca/mortalidade; Hospitalização; População; China; América Latina.
