# Supplementary material for: Escore de Risco Clínico Simples para Prever a Mortalidade Pós Alta Hospitalar em Pacientes Chineses Hospitalizados por Insuficiência Cardíaca
Source: Arq Bras Cardiol. 2023 Feb 13;120(2):e20220250. [Article in Portuguese] doi: 10.36660/abc.2022050 (PMC9972662; doi:10.36660/abc.2022050)
Supplement: Translation

# Translation: A Simple Clinical Risk Score to Predict Post-Discharge Mortality in Chinese Patients Hospitalized with Heart Failure

**Authors:** Guillermo Alberto Arana Morales, Hugo Alpaca-Salvador, Ricardo Salazar-Ramírez

**Affiliations:** 1 Peru Universidade Nacional de Santa, Chimbote - Peru

**Keywords:** Heart Failure/mortality, Hospitalization, Population, China, Latin America

We have read with great interest the article published by Wang et al.^1^on the clinical risk score to predict mortality in Chinese patients hospitalized for heart failure (HF). The authors developed a scale with five variables to predict mortality from HF after hospital discharge within one year: age, female sex, New York Heart Association (NYHA) score >3, left atrial diameter, and body mass index; with good predictive ability.^1^One limitation of the study is the design of random division into two groups, one for the development of the predictive model and the other to evaluate its performance, which, when drawn from the same cohort, have similar characteristics and therefore the predictive performance may be overestimated.^2^

A very interesting aspect of the study is that it focuses on the evaluation of patients with HF at hospital discharge, uses simple, easily assessable predictive variables, and predicts mortality at one year. There are a variety of studies of prognostic scores for HF, in outpatient, hospitalized, or emergency patients, and they predict mortality during hospitalization, within the first 30 days, or a year; many of them with biases that limit their validity.

A systematic review evaluated prognostic models in patients with acute HF in the emergency room. Eight studies were identified. The most frequently estimated predictive outcome was 30-day mortality. The scales evaluated the following predictors: age, NYHA class, systolic pressure, diastolic pressure, oxygen saturation, pCO2 (partial pressure of carbon dioxide), creatinine, atrial natriuretic peptide. Only two scales evaluated were very precise and duly validated.^3^

Iwakami et al. evaluated the performance of existing prognostic scores in a cohort of Japanese patients with acute HF to predict 30-day mortality. It evaluated the characteristics of the derivation study and applied tools to determine biases. Of 6,340 items identified, he studied 224 models. Only 30 (13%) reported c-statistic in the derivation cohort. When the identified models were applied to the Japanese cohort, an overall c-statistic of 0.64 was found. The study found a good correlation between low risk of bias in sample selection and c-statistic. It concludes that an optimal sample in the derivation study is key to determining the performance of HF prognostic models.^4^

Can the predictive model proposed by Wang et al.,^1^or other models be applied to patients in a Latin American population? Sprockel et al.^5^evaluated three existing predictive models for estimating in-hospital death in patients with acute HF from a hospital in Colombia; the ADHERE, OPTIMIZE-HF and GWTG-HF models were evaluated. The study concludes that the risk scales evaluated showed a poor ability to discriminate the risk of intra hospital death.^5^

Studies in Latin America of external validity are definitely required to evaluate the performance, stability and reproducibility of predictive models applied in other geographical areas or, better still, carry out studies to develop and validate their own models.
